# Estimating the Economic Burden of Low Health Literacy in the Blacktown Community in Sydney, Australia: A Population-Based Study

**DOI:** 10.3390/ijerph18052303

**Published:** 2021-02-26

**Authors:** Wadad Kathy Tannous, Moin Uddin Ahmed, James Rufus John, Graham Reece, Golo Ahlenstiel

**Affiliations:** 1 School of Business, Western Sydney University, Parramatta, NSW 2150, Australia; 2 Translational Health Research Institute, Western Sydney University, Campbelltown, NSW 2560, Australia; moin.ahmed@westernsydney.edu.au (M.U.A.); James.John@westernsydney.edu.au (J.R.J.); 3 Intensive Care Unit, Blacktown Hospital, Blacktown, NSW 2148, Australia; graham.reece@health.nsw.gov.au; 4 School of Medicine, Western Sydney University, Penrith, NSW 2751, Australia; golo.ahlenstiel@health.nsw.gov.au

**Keywords:** low health literacy, economic burden, cost, Australia

## Abstract

Evidence shows that inadequate or low health literacy (LHL) levels are significantly associated with economic ramifications at the individual, employer, and health care system levels. Therefore, this study aims to estimate the economic burden of LHL among a culturally and linguistically diverse (CALD) community in Blacktown: a local government area (LGA) in Sydney, Australia. This study is a secondary analysis of cross-sectional data from publicly available datasets, including 2011 and 2016 census data and National Health Survey (NHS) data (2017–2018) from the Australian Bureau of Statistics (ABS), and figures on Disease Expenditure in Australia for 2015–2016 provided by the Australian Institute of Health and Welfare (AIHW). This study found that 20% of Blacktown residents reported low levels of active engagement with health care providers (Domain 6 of the Health Literacy Questionnaire (HLQ)), with 14% reporting a limited understanding of the health information required to take action towards improving health or making health care decisions (Domain 9 of the HLQ). The overall extra/delta cost (direct and indirect health care costs) associated with LHL in the Blacktown LGA was estimated to be between $11,785,528 and $15,432,239 in 2020. This is projected to increase to between $18,922,844 and $24,191,911 in 2030. Additionally, the extra disability-adjusted life year (DALY) value in 2020, for all chronic diseases and age-groups—comprising the extra costs incurred due to years of life lost (YLL) and years lived with disability (YLD)—was estimated at $414,231,335. The findings of our study may enable policymakers to have a deeper understanding of the economic burden of LHL in terms of its impact on the health care system and the production economy.

## 1. Introduction

The health care system is continually evolving—shifting from a “paternalistic model”, where health care providers make decisions for patients, to a “patient-centred model”, where patients are empowered to actively make decisions about their health and health care in order to receive the best possible quality of medical attention [1,2]. In today’s society, there is an increased need for individuals to be “health literate” in order to navigate complex health care systems and understand intricate health information [3]. In 2014, the Australian Commission on Safety and Quality in Health Care (ACSQHC) defined health literacy (HL) as the skills, knowledge, motivation, and capacity of a person to access, understand, appraise, and apply the information necessary to make effective decisions about health and health care and to take appropriate action [4]. Assessing HL allows researchers, health care professionals, and policy makers to appreciate the current ability of community members to understand the information available about their health care needs and to apply this information in order to make informed health decisions that affect positive outcomes [5].

Evidence has shown that HL is the greatest factor influencing health—more than other social determinants, such as education, employment, socioeconomic status, and other lifestyle factors [6,7]. HL mediates the relationship between sociodemographic and socioeconomic factors, thus affecting an individual’s overall health status, health-related quality of life (HRQoL), health behaviours, and use of preventive services [8]. Studies show that patients with greater levels of HL report better clinical and hospital outcomes [9,10]. On the contrary, low HL (LHL)—or, in other words, “health illiteracy”—is associated with significant health burdens and adverse outcomes at both patient and practice levels. At the patient level, studies have shown that LHL is associated with worse health outcomes, poorer HRQoL, and an increased risk of mortality [11,12,13,14]. A systematic review by DeWalt et al. in 2004 [15] estimated that people with LHL are 1.5 to 3 times more likely to experience a poor health outcome. At the practice level, studies have shown that LHL is often associated with non-adherence to taking medication as prescribed or following recommended treatment regimens due to the inability to understand health information [16,17]. This inability to understand health information, coupled with other cultural, social, and systemic barriers, limits both a patient’s capacity to self-manage their medical condition(s) and their health providers’ ability to deliver an appropriate level of care [18].

In Australia, a nationwide Adult Literacy and Life Skills (ALLS) survey in 2006 reported that approximately 60% of Australians had inadequate levels of HL [19]. The Health Literacy Survey (HLS)—which replaced the ALLS survey in 2018—differs from its predecessor by using a conceptual model to capture a wider range of HL domains, including: Australians’ use of health information; their engagement with health providers; and their ability to self-manage medical conditions [20]. The findings of the HLS survey indicated that only one in four Australians strongly agreed that they had sufficient information to manage their health and only 18% strongly agreed that they could actively manage their health [20]. Lower rates of confidence in the ability to manage one’s health were reported by those with chronic health conditions: only 12% of this demographic felt that they had adequate levels of self-management behaviours and only 17% reported active engagement with a health care team [20].

With the ongoing advancements and prevalence of information technology, Australians are often seeking health information online [21]. An Australian cross-sectional study in 2015 indicated that the information available on over 250 Australian health websites was above the average Australian’s level of HL and comprehension [22]. This finding warrants the prioritisation of programs to provide basic HL education to the general public and the development of policies that acknowledge the barriers to health information accessibility. It is imperative that health information be catered to an individual’s level of understanding whilst also being culturally appropriate.

While LHL has been associated with poorer health outcomes, studies have also shown a significant correlation between LHL and economic ramifications at the health care system level [23,24]. A 2007 report released by the University of Connecticut estimated the economic burden of LHL on the United States (US) economy as between $106 billion and $238 billion annually, a figure representing between 7 and 17% of all personal health care expenditure [25]. The first systematic review of the cost of LHL to the health care system by Eichler et al. (2009) indicated an additional fiscal burden of 3 to 5% of the total health care expenditure per year [26]. Eichler et al.’s study also reported the cost of LHL to the individual as ranging from $143 to $7798 per year, compared to those with an adequate level of HL [26]. In Australia, there is currently a knowledge gap with respect to the economic and health care system burden of LHL, with limited studies having been undertaken at the state and national level, and even less at the local level. This study aims to address this knowledge gap by estimating the economic burden of LHL among a culturally and linguistically diverse (CALD) community in Blacktown—a local government area (LGA) in Sydney, Australia.

The article opens with an overview of the materials and methods used in the study—detailing the study design, data sources, source population, and costing methodology—before presenting the research findings and examining their significance at the individual, employer, and health care system levels.

## 2. Materials and Methods

### 2.1. Aims and Objectives

The hypothesis of the study is that LHL is associated with additional costs to different economic agents. The broad aim of this study was to estimate the overall extra or delta cost (direct and indirect health care costs) associated with LHL among residents of the Blacktown LGA.

The specific objectives of the study are as follows:

1. To estimate the cost of LHL to the health care system, the government, employers/businesses, and at the individual and household levels.
2. To estimate the cost of LHL by age group area of health service, and by chronic health condition.
3. To estimate the burden of disease associated with LHL.

### 2.2. Study Design and Data Sources

This study is a secondary analysis of cross-sectional data from publicly available datasets, including 2011 and 2016 census data and National Health Survey (NHS) data of 2017–2018 from the Australian Bureau of Statistics (ABS), and figures on Disease Expenditure in Australia for 2015–2016 provided by the Australian Institute of Health and Welfare (AIHW). The 2017–2018 NHS is the most recent in a series of Australia-wide health surveys conducted by the ABS [27]. The NHS dataset includes data on demographics, socioeconomic factors, chronic conditions, and HL items. The census data were used to forecast the population by age for the Blacktown LGA for 2020. In addition to using publicly available data from the AIHW on Disease Expenditure in Australia for 2015–2016, a literature review was conducted in order to locate suitable research studies and reports to use as the source for the various elements of our cost data.

The study population included people aged over 20 years who were residents of the Blacktown LGA. Extra costs were estimated for the economic burden of LHL for those with chronic diseases.

### 2.3. Source Population

The Blacktown LGA is situated approximately 35 kilometres west of the Sydney central business district, in the state of New South Wales (NSW), Australia [28]. The city spreads across 247 square kilometres and includes 48 suburbs [29]. According to the 2016 census, the Blacktown LGA has the second highest population among LGAs in NSW, comprising 370,000 residents. This is projected to increase to 522,000 by 2036 [29,30]. The Blacktown LGA also has one of the largest urban Aboriginal and Torres Strait Islander populations in Australia, making up 3.2% of the indigenous Australian population [30]. Additionally, the proportion of Blacktown’s CALD community is higher than the NSW average, with 45.9% of residents born in a country other than Australia [28,29]. Compared to the NSW average, the Blacktown LGA has lower Socioeconomic Indexes for Areas (SEIFA) scores (1002 vs. 986), lower levels of tertiary qualifications (23.4% vs. 22.2%), and increased rates of unemployment (6.3% vs. 7.8%) [30].

With respect to the health profile of Blacktown LGA residents, chronic diseases—such as cancer, cardiovascular disease (CVD), respiratory disorders, and mental illness—were reported by the NHS in 2018 to be the leading causes of death across the Blacktown LGA [29,31]. Blacktown LGA residents also report lower indicators of Health-adjusted Life expectancy (HALE), at both birth and at 65 years, compared to the NSW average. In terms of HRQoL and disability, 16 in 100 residents, aged 15 years and above, self-rated their health as fair or poor in the NHS, which again is higher than the average figures for NSW and Australia (14 per 100 people). In addition, the Blacktown LGA has a significantly larger percentage of people living with disability—especially amongst elders aged 85 years and over—compared to Greater Sydney areas (59.8% vs. 50.4%) [29].

In terms of health service utilisation—with the exception of musculoskeletal conditions—the overall hospital admission rates, as well as hospital admissions specific to chronic conditions, were higher among the Blacktown LGA compared to the NSW average [31]. Additionally, modifiable risk factors, such as potentially avoidable hospitalisations, maternal smoking, obesity-related deaths, and hospitalisations, were also higher in comparison to the NSW average [31]. With respect to preventative health service use, the Blacktown community had lower participation rates of bowel, breast, and cervical cancer screening programs compared to the NSW average and other Greater Sydney communities [32].

### 2.4. Health Literacy Questionnaire

The Health Literacy Questionnaire (HLQ) is a widely used multi-dimensional measure of HL developed in 2013 by Osborne et al. in Victoria, Australia [33]. This questionnaire was designed to identify health inequality, inform policy changes, and evaluate HL levels [33]. It has been reported that the HLQ has been used in many countries and in many different settings, including for population health surveys [20,34], the development of interventions [34], and in the evaluation of health programs [35,36,37]. The validity of the HLQ has been widely tested in both English speaking [38,39] and non-English speaking settings [40,41].

Comprising nine domains and 44 items, the HLQ targets independent measures of HL, such as: how a person finds, perceives, and applies health information to effectively manage and make decisions about their health and medical care [33]. Data are collected for each item and domain, based on the respondent’s level of agreement (“strongly agree”, “agree”, “disagree”, or “strongly disagree”) with a set of HL statements or the perceived difficulty of a HL characteristic (“always easy”, “usually easy”, “sometimes difficult”, “usually difficult”, or “cannot do or always difficult”) [29]. It is important to note that all nine domains of the HLQ are considered separate and results are not to be combined across domains [12].

The multidimensional measure of the HLQ survey does not allow for the combination of scores across domains. This study selected Domains 6 (engagement with health care providers) and 9 (understanding health information to take appropriate action, such as seeing a medical practitioner) as they were identified to be the most frequently used measures of HL—as pertains to the self-management of medical conditions—when compared to other domains [42,43] in international research studies. Domain 9 was also chosen due to Osborne et al.’s (2013) finding that this domain of the HLQ was highly correlated with other validated instruments, such as the Test of Functional Health Literacy in Adults (TOFHLA) and the Newest Vital Sign (NVS) health literacy assessment tool [33].

Subscales within each domain were scored from one to five based on difficulty (from “very difficult” to “always easy”). However, as there were no specific cut-off scores provided by the instrument to effectively distinguish between poor and sufficient HL level, we followed a number of computational iterations and used different cut-off scores in our effort to categorise HLQ respondents as possessing an inadequate level of HL if their score was >3.5 or an adequate level of HL if their score was <3.5. These cut-off scores closely resemble the findings on chronic health conditions reported by the NHS of 2017–2018 [20]. Based on the cut-off score, the percentage of respondents with LHL (calculated using Domains 6 and 9 in the HLQ) was calculated across different age groups and applied to the total population of the Blacktown LGA in order to obtain the number and weighted percentage of people with LHL.

### 2.5. Costing Methodology

The costs of health illiteracy were considered for different economic agents, including the public health care provider, individuals, employers, and the government. The economic costing included the cost to Australia’s health care system (Medicare), to households and individuals, employers/businesses, and to the government.

#### 2.5.1. Costs to the Health Care System

Costs to the health care system include extra hospitalisation costs, emergency department visit costs, physician fees, costs for diagnostic tests, pharmaceutical costs due to increased health care need (as a consequence of health illiteracy), and staff turnover costs.

To determine the population for the Blacktown LGA in 2020 by age and gender, the latest available Australian census data of 2011 and 2016 [44,45] were used to calculate the population growth rate of this LGA. The estimated growth rate was then applied to predict the population by age in 2020. Once the total population was determined, the information on HL—obtained from the NHS of 2017–2018—was used to calculate the proportion of people with LHL. The percentage of LHL was multiplied by the total Blacktown LGA population to determine the total population with LHL in this LGA.

We have used the AIHW’s disease expenditure data of 2015–2016—a measurement of the health costs of those who live with chronic conditions—in order to estimate the extra direct costs borne by chronic disease sufferers with LHL. These figures were inflation-adjusted to reflect the 2020-dollar value before the per capita cost was calculated from the expenditure. Following Friedland et al.’s (1998) estimate [46], it was assumed that the extra cost due to LHL is the existing cost, marked-up by 10%. The extra cost was then multiplied by the Blacktown LGA population in order to calculate the total extra cost to different age groups, disease conditions, and service areas. For chronic illness sufferers, the extra cost of LHL was estimated across several areas of medical service, including the services performed by allied health practitioners and general practitioners, the economic strain further prescribed medications place on the pharmaceutical benefits scheme, the additional charges incurred by extra visits to the emergency department of a public hospital, admittance as a patient, and outpatient charges, as well as specialist service providers. Cardiovascular disorders, musculoskeletal disorders, mental illnesses, cancers, chronic kidney diseases, respiratory disorders, and diabetes were the chronic health conditions considered in this study.

**Figure 1:**
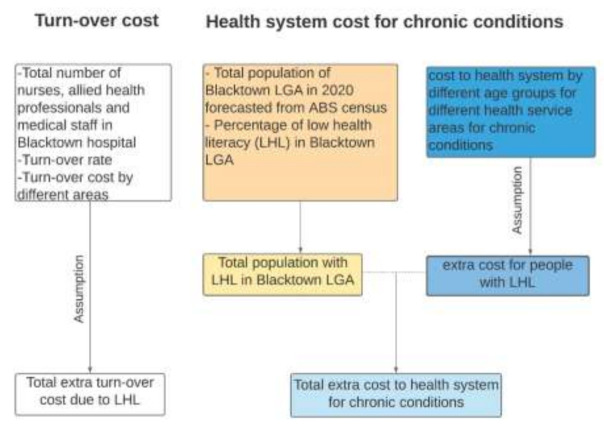
Estimating the economic burden of low health literacy (LHL) on the health care system.

Those delivering health care services face increased stress and professional responsibilities due to the increased demand for health care that LHL levels produce. Research has shown that the turnover rate of health care providers is often high in areas with significant rates of LHL among community members [47]. In this study, we have estimated the extra economic cost of higher rates of staff turnover for nurses, allied health professionals, and other medical staff members (a category that includes staff not classified as nurses, doctors, or other specialists). These staff members comprise about 70% of the total staff in the Western Sydney Local Health District [48]. The total number of nurses, allied health professionals, and other medical staff members at Blacktown Hospital was sourced from a 2015 report by the Western Sydney Local Health District [48]. The population growth rate was applied to this figure in order to estimate the number of staff in 2020. The turnover rate of 10.7% [48] was applied to estimate the full-time staff member turnover in 2020. Our calculations assumed that 10% of this staff turnover was due to LHL. The extra cost of higher rates of staff turnover was calculated by multiplying 10% of the total number of staff turnover with relevant associated costs, such as advertising, hiring, temporary replacement, and training. The figures used to cost the turnover of medical staff were sourced from a 2015 study by Roche et al. that estimated the cost of nurse turnover in Australia and adjusted for inflation [49]. We have assumed that the costs for allied health professionals and other medical staff members are similar to nurses. Our framework for estimating the health care system cost is summarised in Figure 1.

#### 2.5.2. Costs to Individuals, Households, and Carers

**Figure 2:**
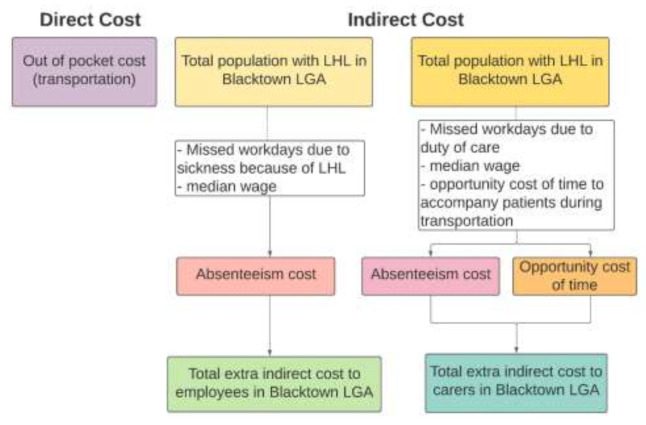
Estimating the economic costs of LHL to individuals and households.

Studies have shown that people with LHL tend to experience poorer health outcomes than those with higher rates of HL (Smith et al., 2018; Tormey et al., 2019; Panagioti et al., 2018; González-Chica et al., 2016), which results in both financial and non-financial burdens. The present study considered transportation fees for extra visits to physicians and hospitals direct out-of-pocket costs for those with LHL. To calculate indirect costs at the individual level, two components were considered: absenteeism costs and presenteeism costs. An absenteeism cost pertains to the loss of income an employee experiences when they are unable to attend work. Absent days are converted into weeks and multiplied with the median weekly wage to estimate the cost of absenteeism. In addition to assuming that those with LHL will miss a greater number of workdays than those with adequate levels of HL, we have also assumed that their likely lower health status will affect their performance at work. Their progress may be much lower than those employees without any medical conditions. This is considered as a presenteeism cost at the individual level. The conceptual framework we have used for calculating the production loss cost due to LHL is summarised in Figure 2.

The opportunity cost of carers’ time is also calculated as adding to the overall financial burden of LHL (Figure 2). The opportunity cost for carers is the value of their time that is lost as the result of having to take care of someone with an LHL-related health condition. If the carer was not engaged in looking after the individual with LHL, they could have participated in paid work. The relevant wage rate and number of missed days/time spent performing a carer’s role were multiplied to find the LHL burden for carers.

#### 2.5.3. Costs to the Employer/Business

**Figure 3:**
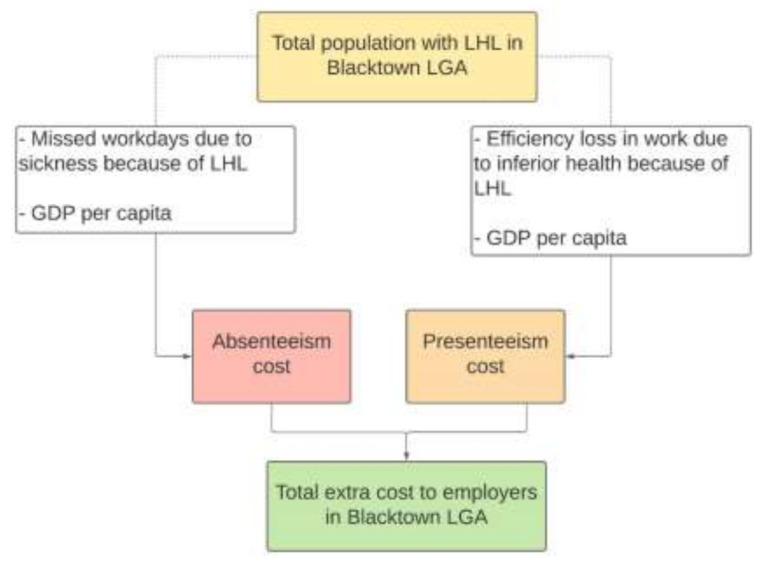
Estimating the economic cost of LHL to the employer/business.

Employers bear costs due to LHL in two ways. Since people with LHL are more likely to suffer from chronic conditions, this typically results in more days absent from work than average. Therefore, an employer’s output and revenue will be less than their potential as the result of an under-utilisation of labour resources and the payment of full or partial salaries to their employees who may frequently be absent from work. Presenteeism cost as the result of LHL-related medical conditions has also been estimated and costed as a relevant factor producing lower production rates and, therefore, lower profits (Figure 3).

#### 2.5.4. Costs to the Government

**Figure 4:**
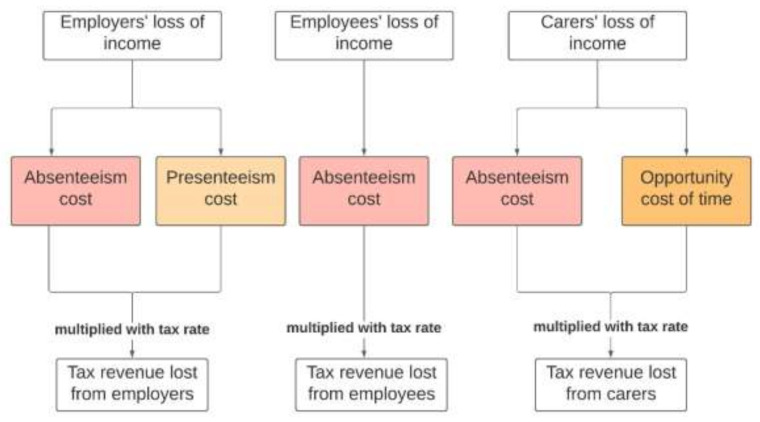
Estimating the economic cost of LHL to the government.

The government also experiences the financial burden of LHL in terms of lost tax revenue. Once the cost of the individual’s forgone salary, carers’ costs, and production losses are calculated, this figure can be used to estimate the total lost tax revenue. The average tax rate was multiplied with the annual figure to estimate the loss of tax revenue for the government (Figure 4).

#### 2.5.5. Burden of Disease Due to LHL

A non-financial aspect of the cost of LHL is people’s experience of pain, suffering, and increased probability of early mortality. This burden is estimated in terms of Disability-adjusted life years (DALYs): a measure of the overall burden of a disease that takes into consideration years lived with a disability (YLD) and years of life lost (YLL) as the result of early death. These non-financial burdens are converted into monetary terms through using the value of a statistical life year estimate—the Australian Government currently estimates this as $213,000 [50]—in order to reflect the financial burden of LHL-related disability to the individual. The latest available Australian Burden of Disease Study data (2015) [51] were used to find out the burden of disease as the result of LHL for the Blacktown LGA. In order to calculate the burden of different diseases, YLD and YLL were sourced from the Australian Burden of Disease Study data of 2015. The proportion of the Australian population living in the Blacktown LGA was obtained for different age groups by using the ABS census data of 2011 and 2016. Assuming that the burden is shared according to the above proportion, the total YLD and YLL of different diseases for Australia were multiplied with the Blacktown LGA’s percentage of the population. This figure was then multiplied by 5% of the Blacktown LGA’s community that suffers from chronic disease(s) (5% being our assumption of the percentage of disabilities that are influenced by LHL) in order to obtain each component of DALY. The calculated DALY was multiplied with the value of statistical life-years (VSLYs) and adjusted for inflation.

## 3. Results

### 3.1. Prevalence of LHL in the Blacktown LGA

The prevalence of LHL in the Blacktown LGA population in 2020—using figures from Domains 6 and 9 of the HLQ—is shown in Table 1. One in five (20%) residents in the Blacktown community reported low levels of active engagement with health care providers (Domain 6) and 14% reported a limited understanding of the health information required to take action towards health and health care decisions (Table 1).

### 3.2. Total Extra/Delta Direct Health Care Costs

#### 3.2.1. Domain 6—Engagement with Health Care Providers

With respect to engagement with health care providers (Domain 6), the total extra direct health costs associated with chronic diseases were estimated to be $9,166,409. A breakdown of total extra direct health costs by type of chronic disease group is shown in Table 2. Of the chronic disease groups considered in this study, LHL was most strongly associated with additional costs for mental illness treatments—accounting for 25% of the total extra costs for chronic disease sufferers—followed by musculoskeletal disorders (20.6%) and CVD (18.3%) (Table 2). Tertiary care accounted for almost 90% of the additional direct health care costs of individuals with LHL who suffer from chronic diseases.

The additional direct health costs incurred as a result of LHL are shown in Table 3, reporting both the overall cost and cost by area of service. Falling under tertiary care costs, public hospital admissions accounted for almost 35.6% of the total extra costs, followed by an additional financial strain on the Pharmaceutical Benefits Scheme (20.5%) and hospital outpatient services (10.6%). A further breakdown of the total extra direct health costs by area of health service, and separated by each type of chronic disease group, is presented in Appendix A.

In terms of age group, those 45 years and above accounted for more than half of the total extra direct health care costs (65.7%) in comparison to the younger population of the Blacktown LGA (Table 4). This may be explained by the greater prevalence of chronic diseases in persons aged over 45 years.

#### 3.2.2. Domain 9—Understanding Health Care Information in Order to Take Action

With respect to the ability to understand health information and take appropriate action (Domain 9)—such as seeing a medical practitioner—the total extra direct health costs associated with chronic diseases were estimated to be $8,405,342. A breakdown of additional cost by type of chronic disease group is shown in Table 5. Of the chronic disease groups, LHL was most strongly associated with CVD, accounting for 22.2% of the total extra costs, followed by musculoskeletal disorders (20%) and mental illness (18.6%) (Table 5). Tertiary care accounted for more than 90% of the total direct additional costs of LHL for those suffering from chronic disease(s).

The additional direct health costs by area of health service are shown in Table 6. Public hospital admissions accounted for almost 43% of the total extra costs, followed by additional costs to the Pharmaceutical Benefits Scheme (24%), and fees for hospital outpatient services (11%).

Consistent with the higher prevalence of LHL noted among the elderly population in Domain 9 of the HLQ, people aged 65 years and above accounted for more than half of the total extra direct health care costs (53%), as shown in Table 7.

#### 3.2.3. Out of Pocket Costs

The direct costs to the individual include gap fees for health service appointments and transportation costs for extra visits to hospital or physicians. In this study, we have considered the cost of transportation to and from the hospital as an out-of-pocket cost. According to Blacktown Hospital’s reported figures, in 2020, there were 43,376 hospital attendances in the first six months. If we assume the same number of attendances for the second half of the year, then the estimated number of hospital admissions per annum at Blacktown Hospital is 86,752. We have assumed that 10% of these visits are repeated visits due to LHL-related health conditions. Looking at the ABS census data of 2016, 7% of residences in the Blacktown LGA did not have a registered motor vehicle (we are assuming that most of the patients attending Blacktown Hospital are from the Blacktown LGA), meaning that 607 patients would have been admitted after having travelled by taxi, with the remainder travelling to hospital by using their own car. We calculated the transportation costs of travelling to Blacktown Hospital via taxi as $54,654 per annum (assuming that transportation will be needed both ways and using the average taxi fee of $45.00 per trip for our estimation). We have also estimated transportation costs for emergency department (ED) visits in a similar way. There were 96,405 visits to the ED of Blacktown Hospital in 2019. Taking population growth into account, we estimated that the number of ED visits for 2020 totalled 98,782. We estimated the annual transportation costs of using taxis for ED visits as $62,233. The total overall out-of-pocket transportation cost for LHL-related hospital visits was estimated as $116,886.

### 3.3. Total Extra/Delta Indirect Health Care Costs

Total indirect costs include costs to the employer, the individual, carers, and the government (Table 8). The total indirect additional cost due to LHL in the Blacktown LGA was estimated as $3.1 million if one week of work was missed due to poor health. It may be double that, at almost $6 million, if 10% of working days in the year are missed.

#### 3.3.1. Costs to the Employer/Business

We have calculated both types of costs for employers: the absenteeism cost and the presenteeism cost. In order to do this, we first calculated the total number of people in the labour force in the Blacktown LGA. This figure was 258,223 for those aged 20 years and over in 2020. Given that 13.9% (domain 9) of people in this LGA have LHL, we have calculated the total number of people with LHL as 35,667. We have assumed that labour force participation rate does not vary by level of HL. The Blacktown LGA’s specific labour force participation rate of 60.6% and the employment rate of 91.9% were used to calculate the total number of employed people with LHL. We estimated that the total number of employed people with LHL was 19,864 for the Blacktown LGA in 2020. We also used this figure to calculate the absenteeism and presenteeism costs.

The absenteeism cost requires the total number of additional workdays missed due to an LHL-related sickness. To find out the total number of extra days missed due to illness, we have used the ABS NHS data of 2017–2018. Those with LHL are absent 1.42% more than those with adequate levels of HL, creating an additional absenteeism cost to the employer [20]. This extra cost was calculated by multiplying the extra 1.42% with the total number of employed people with LHL in the Blacktown LGA (*n* = 19,864). The resulting figure was multiplied with GDP per capita (converted into a weekly figure) in order to calculate the total monetary value of production loss due to LHL-related absenteeism. If we assume that employers only pay for missed workdays for their full-time employees—61.1% in the Blacktown LGA—then the total loss due to absenteeism is calculated as $281,421 per annum. This estimated loss increases to $1.5 million if absent days equate to 10% of workdays in a year (Table 8).

The calculation of the presenteeism cost of LHL is based on some assumptions. We have assumed that 50% of the total number of employees with LHL is 5% less efficient than those with adequate HL levels. We have also assumed that this inefficiency occurs for 5% of the total weeks in a year. The resulting loss in production is estimated as $1.8 million (Table 8). In our estimates, the total costs of absenteeism and presenteeism to the employer as the result of LHL can vary from $2.1 million to $3.3 million (Table 8).

#### 3.3.2. Costs to Individuals and Households

The indirect costs to the individual due to LHL come in many different forms. Individuals bear the cost of absenteeism in terms of loss of wages. There is also a presenteeism cost caused by inefficiency at work. Since those with LHL are likely to have a lower health status on average, this will affect their performance at work. Their progress is likely to be much lower than it would be if they were in full health. Carers of sick individuals also suffer financial losses in terms of lost wages. In this report, we have considered the absenteeism cost borne by the employees and the cost to carers in needing to provide support to them. We have not calculated the presenteeism cost to individuals due to the nature of its complexity in modelling and the inability to source adequate data. We have assumed that employees with part-time or casual employment will lose their salaries for missed workdays due to illness. In order to find out the annual cost of absenteeism to part-time employees, we multiplied the total number of part-time employees with LHL who missed work with the median wage of $658 (inflation-adjusted), arriving at an estimated cost of $56,777 (Table 8). This cost can increase to $296,037 (Table 8) if missed workdays comprise 10% of the total weeks in a year.

We have calculated the cost to carers by assuming that they provide care for one-third of the period that individuals with LHL are absent from work. Forgoing wages in providing care, the estimated annual cost to carers varies from $188,552 to $1 million (Table 8). Carers also incur an opportunity cost in the form of leisure time that is consumed in helping those with LHL travel to the hospital for appointments or treatments or to visit the ED as well as time spent waiting in the ED. If we assume the opportunity cost of carers’ time to be 30 min each way (to and from the hospital), then this translates to a $30,363 per annum additional cost for hospital visits. Furthermore, the opportunity cost to carers when visiting and waiting in the ED is estimated as $149,358 per annum. Therefore, the total indirect cost to carers for waiting and visiting the hospital and the ED is $179,722 per year.

#### 3.3.3. Costs to the Government

Lost tax revenue constitutes the indirect costs incurred by the government due to LHL. This lost revenue is calculated by multiplying the average tax rate of 30% with the total lost value of production. In our calculations, the estimated annual tax revenue loss varies from $711,595 to $1.4 million (Table 9).

### 3.4. Burden of Disease Due to LHL

The overall extra burden of disease cost associated with LHL in the Blacktown LGA is presented in Table A11. The extra DALY value (in 2020) for all chronic diseases and age-groups is estimated as $414,231,335, which includes the additional costs of YLL ($196,687,229) and YLD ($217,544,106)

## 4. Discussion

This study explored the prevalence of LHL in the CALD community of the Blacktown LGA and its associated economic costs. Our findings demonstrate that 20% of Blacktown LGA residents reported low levels of active engagement with health care providers in the HLQ survey (Domain 6) and that 14% had a limited understanding of the health information required to take action towards improving health or making health care decisions. With respect to age, the younger population of this LGA—those between 20 and 34 years—reported lower levels of engagement with health care providers and a limited understanding of health information compared to the elderly population. This may be due to the fact that younger adults are more likely to be healthy and, as a result, less likely to be sourcing information on health, or medical care, compared to those who are chronically ill and/or elderly [52,53,54].

Previous studies—particularly research from the US—have examined the economic burden of LHL. In Australia, while the prevalence of LHL has been discussed at the national level, the additional health care cost associated with LHL has not been explored. This is the first study in Australia to estimate the extra costs associated with LHL among the Blacktown LGA community. A scoping review by Choudhry and his colleagues [55] supports this claim that there are no comparable cost figures for Australia. The estimated costs of LHL-related health care were calculated across several areas: costs to the health care system, productivity costs to the employer, transactional costs, out-of-pocket costs to the individual, and the costs of the burden of disease. The overall extra/delta cost (direct and indirect health care costs) associated with LHL among the Blacktown LGA community was estimated to be between $11,785,528 and $15,432,239 in 2020. This is projected to increase to between $18,922,844 and $24,191,911 in 2030. The additional DALY value (in 2020) of LHL for all chronic diseases and age-groups was estimated as $414,231,335.

To compare our cost figures to other countries, we have calculated the per capita cost and its share of the health expenditure. The per capita cost due to LHL in the Blacktown LGA for those aged 20 years and above with chronic conditions was estimated to be between $32.5 and $35.5 per year. The per capita health expenditure in Australia in 2018–2019 was $7772 [56], indicating that 0.5% of the health expenditure for this period was due to LHL. The systematic review by Eicher and colleagues [26] found that additional expenditure per year due to LHL, at the patient level, varies from USD 143 to USD 7798. Two studies, for instance, found remarkably different prices for overall care and inpatient care: (USD 7798) [57] compared to (USD 1551) [58] for the former, and (USD 6214) [57] compared to (USD 1543) [58] for the latter. Price differences were also noted for outpatient care and hospital-based care [59]. Additional health system costs ranged from 3 to 5% of the total health care cost. Another study by Haun and co-authors [60] suggested that LHL is associated with up to USD 14,548 per patient for three years—an estimate gathered from a sample of veterans in the North Florida/South Georgia region.

Estimates from our study were lower than what has previously been found. One reason for this can be that our study takes a conservative approach to estimate the additional costs incurred due to LHL. Other studies have also been quite heterogeneous in their methodology. The methodology of our study is different from earlier studies in terms of our population, decision to include chronic health conditions in our costing, and the areas of health care that we focused on. It is also a challenge to accurately transfer the cost implications from studies conducted in other countries and apply them to the Australian health care system.

### 4.1. Strengths and Limitations

This is the first study in Australia to estimate the additional health costs associated with LHL in the Blacktown LGA community. This study also draws strength from using nationally representative datasets sourced from surveys and ABS censuses. The estimation of extra or delta health care costs has been stratified according to the type of chronic disease group, area of health service, and age group. In addition, extra costs incurred due to LHL have been calculated from different agents’ perspectives (the individual and household, employee, employer, and the government).

This study, however, has several limitations. It does not include gap fees as part of out-of-pocket costs to the individual or the associated presenteeism cost of LHL to the employee. We have not considered calculating the presenteeism cost to the individual due to the nature of its complexity in modelling and the data requirement. Annual staff turnover costs as a result of LHL were also not calculated in the study. Recognition has been made that the health illiteracy of patients causes additional stress for hospital staff members, creating the potential for greater staff turnover. However, we were unable to determine the turnover costs associated with LHL as the average turnover of Western Sydney Local Health Distinct (WSLHD) staff was comparable to several local health districts (LHDs) across NSW at a rate of 10.7% [48].

Although this study found a direct correlation between LHL levels and additional health care costs, it is important to note that HL and LHL levels are not purely symmetric: that is, increasing HL levels is not a guarantee of reducing the economic burden associated with LHL. Rather, LHL levels are but one factor placing an economic strain on the individual, employer, and health care system and, as such, a multifaceted approach would be required.

### 4.2. Policy Implications and Future Research

The findings of our study may enable policymakers to have a deeper understanding of the economic burden of LHL in terms of its impact on the health care system and the production economy. This research may also help clarify who bears the burden of LHL, aiding the efficient allocation of resources to alleviate the cost of LHL to the individual, public health care provider, and the government. With respect to future research, there is an opportunity to delve into all nine domains of the HLQ in order to obtain a deeper understanding of LHL and evaluate the costs associated with the different aspects of LHL. Once data on Primary Health Network (PHN) levels are available, future research could focus on comparing the Western Sydney PHN population to that of neighbouring PHNs (South Western and Nepean Blue Mountains PHNs) as well as high performing and affluent PHNs (North Sydney PHN).

## 5. Conclusions

Inadequate levels of HL have economic ramifications for individuals, employers, providers of health care services, as well as for wider society. This is the first study in Australia to estimate the extra costs associated with LHL in the Blacktown LGA community. The extra cost incurred due to LHL was explored from different agents’ perspectives (individual and household, employee, employer, and government). It is imperative that prevention and management initiatives be introduced to reduce the impact of LHL both for the individual and the wider community. Policies that focus on implementing educational programs to help raise awareness and HL levels, especially among CALD communities, may help alleviate the costs associated with LHL.

## Figures and Tables

**Table 1:** The prevalence of LHL in the Blacktown local government area (LGA) by domain (6 and 9).

Age Groups	Total Population 2020 (*n* = 258,223)	LHL Population (Domain 6) (*n* = 53,451)	% of LHL (Domain 6)	LHL Population (Domain 9) (*n* = 35,667)	% of LHL (Domain 9)
20–24 years	24,368	12,050	49.45	3979	16.33
25–34 years	56,171	2663	4.74	2663	4.74
35–44 years	55,807	13,667	24.49	6741	12.08
45–54 years	44,218	14,888	33.67	10,573	23.91
55–64 years	36,522	6428	17.60	2410	6.6
≥65 years	41,137	3756	9.13	9301	22.61
Prevalence of LHL			20.70		13.81

**Table 2:** Total extra direct health care costs incurred due to LHL by type of chronic disease group in the Blacktown LGA (Domain 6).

Type of Chronic Disease Group	Direct Health Care Cost ($)	Direct Health Care Cost (%)
Cardiovascular disorders	1,680,869	18.3
Musculoskeletal disorder	1,886,681	20.6
Mental illness	2,269,347	24.8
Cancer	1,298,083	14.2
Chronic Kidney Disease	665,277	7.3
Respiratory disorders	705,970	7.7
Diabetes	660,182	7.2
Preventive care (sub-total)	952,366 (10.4%)	
Tertiary care (sub-total)	8,214,043 (89.6%)	
Total	9,166,409	100.0

**Table 3:** Additional direct health care costs incurred due to LHL for those with a chronic condition (by area of health service) in the Blacktown LGA (Domain 6).

Area of Service	Direct Health Care Cost ($)	Direct Health Care Cost (%)
Allied health and other services	481,270	5.3
Diagnostic services	647,041	7.1
General Practitioner	952,366	10.4
Pharmaceutical benefits scheme	1,879,884	20.5
Public hospital admitted patient	3,263,931	35.6
Public hospital emergency department	401,874	4.4
Public hospital outpatient	970,820	10.6
Specialist services	569,221	6.2
Total	9,166,407	100.0

**Table 4:** Additional direct health care costs incurred due to LHL for those with a chronic condition (by age group) in the Blacktown LGA (Domain 6).

Age Group	Direct Health Care Cost ($)	Direct Health Care Cost (%)
20–24 years	1,003,541	10.9
25–34 years	278,482	3.0
35–44 years	1,858,506	20.3
45–54 years	2,609,297	28.5
55–64 years	1,634,683	17.8
65 years and over	1,781,899	19.4
Total	9,166,408	100.0

**Table 5:** Additional direct health care costs incurred due to LHL (by type of chronic disease group) in the Blacktown LGA (Domain 9).

Type of Chronic Disease Group	Direct Health Care Cost ($)	Direct Health Care Cost (%)
Cardiovascular disorders	1,866,019	22.2
Musculoskeletal disorders	1,680,558	20.0
Mental illness	1,563,722	18.6
Cancer	1,337,973	15.9
Chronic Kidney Disease	661,653	7.9
Respiratory disorders	689,221	8.2
Diabetes	606,196	7.2
Preventive care (sub-total)	768,906 (9.1%)	
Tertiary care (sub-total)	7,636,435 (90.9%)	
Total	8,405,342	100.0

**Table 6:** Additional direct health care costs incurred due to LHL for those with a chronic condition (by area of health service) in the Blacktown LGA (Domain 9).

Area of Service	Direct Health Care Cost ($)	Direct Health Care Cost (%)
Allied health and other services	329,940	4.3
Diagnostic services	535,870	7.0
General Practitioner	768,906	10.1
Pharmaceutical benefits scheme	1,819,236	23.8
Public hospital admitted patient	3,272,324	42.9
Public hospital emergency department	341,529	4.5
Public hospital outpatient	840,620	11.0
Specialist services	496,915	6.5
Total	8,405,342	100.0

**Table 7:** Additional direct health care costs incurred due to LHL for those diagnosed with a chronic condition (by age group) in the Blacktown LGA (Domain 9).

Age Groups	Direct Health Care Cost ($)	Direct Health Care Cost (%)
20–24 years	331,401	3.9
25–34 years	278,482	3.3
35–44 years	916,731	10.9
45–54 years	1,852,934	22.0
55–64 years	613,006	7.3
65 years and over	4,412,787	52.5
Total	8,405,342	100.0

**Table 8:** Additional annual indirect costs due to LHL in the Blacktown LGA.

Type of Agent	Type of Cost	Missed Weeks Due to Sickness	
		1 Week	10% of Weeks in a Year
Employer	Absenteeism cost	281,421	1,467,329
	Presenteeism cost	1,845,233	
	Total	2,126,653	3,312,561
Individual	Absenteeism cost	56,777	296,037
	Opportunity cost of time due to transportation	30,363	
Carers	Carer’s cost	188,552	983,110
Government	Lost tax revenue-employer (absenteeism)	84,426	440,199
	Lost tax revenue-employer (presenteeism)	553,570	
	Lost tax revenue-employer (total)	637,996	993,768
	Lost tax revenue-employee(absenteeism)	17,033	88,811
	Lost tax revenue-carer (absenteeism)	56,566	294,933
	Total	711,595	1,377,513
Total Indirect cost		3,113,940	5,999,584

**Table 9:** Extra burden of disease cost due to LHL in the Blacktown LGA.

Age Groups	Cancer and Other Neoplasms	Cardiovascular Diseases	Endocrine Disorders	Kidney and Urinary Diseases	Mental Illness and Substance Abuse Disorders	Musculoskeletal Conditions	Respiratory Diseases
20–24 years	2.95	1.59	0.26	0	0.21	0	0.46
25–34 years	13.06	7.17	0.85	0.53	0.89	0.39	0.9
35–44 years	37.66	20.7	1.93	1.73	1.18	0.69	2.01
45–54 years	72.95	35.93	3.47	2.56	2.28	1.06	5.55
55–64 years	125.11	49.48	5.35	4.65	1.55	1.73	11.73
65–74 years	139.05	62.78	6.57	5.95	1.36	2.2	23.75
75–84 years	76.01	58.65	5.83	6.38	0.96	1.89	18.39
85 years and over	26.16	52.78	3.33	4.56	0.71	1.48	9.28
Extra YLL for all chronic diseases	492.95	289.08	27.59	26.35	9.15	9.45	72.07
Extra YLL value (2020 $) for all chronic diseases	104,632,769	61,360,061	5,856,250	5,593,916	1,941,598	2,004,835	15,297,799
Extra YLL value (2020 $) for all chronic diseases and age-groups	**196,687,229**						
20–24 years	2.33	1.38	0.82	0.09	31.71	9.39	6.12
25–34 years	10.29	5.97	2.63	0.64	77.38	30.28	13.43
35–44 years	26.19	15.01	4.78	1.43	65.29	39.57	15.19
45–54 years	43.22	22.74	6.47	1.93	37.07	40.96	14.39
55–64 years	63.69	29.37	8.75	2.99	16.59	43.88	16.23
65–74 years	65.89	36.72	8.46	3.7	7.09	36.11	21.3
75–84 years	30.98	28.56	4.02	3.21	1.9	13.18	12.62
85 years and over	9.56	21.07	1.49	1.88	0.68	3.31	4.94
Extra YLD for all chronic diseases	252.17	160.82	37.41	15.88	237.72	216.68	104.23
Extra YLD value (2020 $) for all chronic diseases	53,524,081	34,136,295	7,940,133	3,370,362	50,458,071	45,992,590	22,122,575
Extra YLD value (2020 $) for all chronic diseases and age-groups	**217,544,106**						
20–24 years	5.28	2.97	1.07	0.09	31.92	9.39	6.58
25–34 years	23.35	13.14	3.47	1.18	78.27	30.67	14.33
35–44 years	63.84	35.72	6.71	3.16	66.48	40.26	17.21
45–54 years	116.17	58.67	9.94	4.5	39.36	42.03	19.94
55–64 years	188.81	78.85	14.1	7.64	18.14	45.61	27.96
65–74 years	204.94	99.5	15.03	9.65	8.46	38.31	45.06
75–84 years	106.99	87.21	9.85	9.59	2.86	15.07	31
85 years and over	35.72	73.85	4.82	6.43	1.39	4.79	14.22
Extra DALY for all chronic diseases	745.12	449.91	65	42.23	246.87	226.13	176.3
Extra DALY value (2020 $) for all chronic diseases	158,156,850	95,496,356	13,796,382	8,964,278	52,399,669	47,997,425	37,420,374
Extra DALY value (2020 $) for all chronic diseases and age-groups	**414,231,335**						

## Data Availability

Data sharing not applicable. No new data were created or analyzed in this study. Data sharing is not applicable to this article.

## Appendix A

**Table A1:** Extra costs incurred due to LHL for people diagnosed with *CVD* in the Blacktown LGA (Domain 6).

Age Groups	Allied Health and Other Services	Diagnostic Tests	General Practitioner	Pharmaceutical Benefits Scheme	Public Hospital Admitted Patient	Public Hospital Emergency Department	Public Hospital Outpatient	Specialist Services	Total Services
20–24 years	224	10,966	30,372	4282	33,446	7051	11,685	6477	104,503
25–34 years	71	3813	7703	1870	9301	1911	4029	2013	30,711
35–44 years	609	21,380	46,342	27,686	90,545	17,038	19,709	16,880	240,189
45–54 years	1113	27,534	58,310	82,743	197,814	32,788	28,102	28,519	456,923
55–64 years	851	15,749	30,421	84,154	161,247	21,496	19,546	20,351	353,815
65 years and over	1291	13,342	29,090	120,164	258,808	30,343	22,093	19,595	494,726
Total (inflation adjusted)	4158	92,784	202,239	320,900	751,162	110,626	105,164	93,835	1,680,868

**Table A2:** Extra costs incurred due to LHL for people diagnosed with *Musculoskeletal disorders* in the Blacktown LGA (Domain 6).

Age Groups	Allied Health and Other Services	Diagnostic Tests	General Practitioner	Pharmaceutical Benefits Scheme	Public Hospital Admitted Patient	Public Hospital Emergency Department	Public Hospital Outpatient	Specialist Services	Total Services
20–24 years	2781	31,233	31,108	35,442	34,101	11,837	38,073	5816	190,391
25–34 years	962	9503	7776	13,244	8247	2698	13,701	1799	57,930
35–44 years	8607	63,358	45,045	119,018	65,008	15,912	60,584	13,298	390,830
45–54 years	14,626	91,119	55,511	177,845	120,473	18,253	65,446	19,913	563,186
55–64 years	10,367	53,311	28,876	100,414	93,162	7638	38,683	13,837	346,288
65 years and over	13,159	41,525	28,501	83,714	111,995	6974	38,229	13,960	338,057
Total (inflation adjusted)	50,501	290,049	196,817	529,677	432,986	63,312	254,716	68,623	1,886,681

**Table A3:** Extra costs incurred due to LHL for people diagnosed with *Mental illness* the Blacktown LGA (Domain 6).

Age Groups	Allied Health and Other Services	Diagnostic Tests	General Practitioner	Pharmaceutical Benefits Scheme	Public Hospital Admitted Patient	Public Hospital Emergency Department	Public Hospital Outpatient	Specialist Services	Total Services
20–24 years	95,779	4791	48,415	32,283	182,462	37,153	29,514	26,899	457,296
25–34 years	22,621	1506	11,438	10,297	45,868	6970	9022	6497	114,219
35–44 years	120,839	9102	64,236	81,736	251,151	35,292	54,718	41,693	658,767
45–54 years	111,592	12,399	71,395	95,324	209,393	29,276	47,381	46,621	623,381
55–64 years	40,378	7215	32,721	38,773	72,867	8790	16,368	20,133	237,245
65 years and over	18,212	6049	27,071	24,041	70,118	7660	16,105	9184	178,440
Total (inflation adjusted)	409,421	41,063	255,275	282,453	831,858	125,142	173,109	151,027	2,269,348

**Table A4:** Extra costs incurred due to Low HL for people diagnosed with *Cancer* in Blacktown LGA (Domain 6).

Age Groups	Allied Health and Other Services	Diagnostic Tests	General Practitioner	Pharmaceutical Benefits Scheme	Public Hospital Admitted Patient	Public Hospital Emergency Department	Public Hospital Outpatient	Specialist Services	Total Services
20–24 years	63	5027	8405	5562	18,993	297	11,251	6672	56,270
25–34 years	20	1757	2241	2615	6676	94	3712	2359	19,474
35–44 years	165	12,339	14,794	40,297	64,766	866	31,165	23,650	188,042
45–54 years	304	18,575	22,022	90,097	146,472	2020	67,153	50,909	397,552
55–64 years	224	11,852	13,992	71,737	117,094	1632	54,031	39,709	310,271
65 years and over	323	10,793	15,517	79,212	125,912	1715	54,312	38,691	326,475
Total (inflation adjusted)	1099	60,343	76,970	289,520	479,912	6625	221,624	161,990	1,298,083

**Table A5:** Extra costs incurred due to LHL for people diagnosed with *Chronic kidney disease* in the Blacktown LGA (Domain 6).

Age Groups	Allied Health and Other Services	Diagnostic Tests	General Practitioner	Pharmaceutical Benefits Scheme	Public Hospital Admitted Patient	Public Hospital Emergency Department	Public Hospital Outpatient	Specialist Services	Total Services
20–24 years	31	6633	4239	5051	21,201	6253	6290	1748	51,446
25–34 years	10	2119	1064	1841	6265	1495	2265	551	15,610
35–44 years	86	11,062	6130	17,299	56,188	8801	13,365	4314	117,245
45–54 years	157	13,377	7534	27,306	110,725	11,336	20,726	6474	197,635
55–64 years	120	7439	3923	15,935	77,787	5892	13,471	4299	128,866
65 years and over	182	6030	3840	15,613	109,107	6135	9595	3972	154,474
Total (inflation adjusted)	585	46,660	26,730	83,045	381,274	39,912	65,712	21,359	665,277

**Table A6:** Extra costs incurred due to LHL for people diagnosed with *respiratory disorders* in the Blacktown LGA (Domain 6).

Age Groups	Allied Health and Other Services	Diagnostic Tests	General Practitioner	Pharmaceutical Benefits Scheme	Public Hospital Admitted Patient	Public Hospital Emergency Department	Public Hospital Outpatient	Specialist Services	Total Services
20–24 years	103	4891	17,418	15,199	27,233	7778	8314	4221	85,157
25–34 years	33	1364	4326	4085	5524	1426	2850	1399	21,007
35–44 years	281	9070	25,073	26,650	35,880	7977	12,715	11,205	128,851
45–54 years	514	13,293	30,877	40,498	57,387	11,070	14,125	16,467	184,231
55–64 years	393	7996	16,100	30,004	45,235	7553	8481	10,030	125,792
65 years and over	596	6527	15,682	40,531	69,606	12,245	7900	7842	160,929
Total (inflation adjusted)	1921	43,141	109,477	156,968	240,864	48,049	54,385	51,165	705,970

**Table A7:** Extra costs incurred due to LHL for people diagnosed with *Endocrine disorders* in the Blacktown LGA (Domain 6).

Age Groups	Allied Health and Other Services	Diagnostic Tests	General Practitioner	Pharmaceutical Benefits Scheme	Public Hospital Admitted Patient	Public Hospital Emergency Department	Public Hospital Outpatient	Specialist Services	Total Services
20–24 years	667	8873	12,899	9138	9819	2140	12,977	1962	58,475
25–34 years	217	2960	3240	4700	2839	370	4595	610	19,531
35–44 years	1991	16,817	18,968	46,668	23,341	1868	20,520	4410	134,583
45–54 years	3871	21,772	23,957	59,555	42,445	2038	26,465	6289	186,392
55–64 years	3012	12,255	12,941	51,170	30,588	932	17,377	4129	132,404
65 years and over	3827	10,324	12,852	46,090	36,844	861	14,175	3823	128,796
Total (inflation adjusted)	13,585	73,001	84,858	217,321	145,876	8209	96,109	21,223	660,182

**Table A8:** Extra costs incurred due to LHL for people diagnosed with *CVD* in the Blacktown LGA (Domain 9).

Age Groups	Allied Health and Other Services	Diagnostic Tests	General Practitioner	Pharmaceutical Benefits Scheme	Public Hospital Admitted Patient	Public Hospital Emergency Department	Public Hospital Outpatient	Specialist Services	Total Services
20–24 years	74	3621	10,030	1414	11,045	2328	3859	2139	34,510
25–34 years	71	3813	7703	1870	9301	1911	4029	2013	30,711
35–44 years	300	10,546	22,859	13,657	44,663	8404	9722	8326	118,477
45–54 years	790	19,552	41,408	58,758	140,474	23,284	19,956	20,252	324,474
55–64 years	319	5906	11,408	31,558	60,468	8061	7330	7632	132,682
65 years and over	3196	33,041	72,041	297,581	640,926	75,143	54,711	48,526	1,225,165
Total (inflation adjusted)	4750	76,480	165,448	404,838	906,876	119,131	99,607	88,888	1,866,018

**Table A9:** Extra costs incurred due to LHL for people diagnosed with *Musculoskeletal disorders* in the Blacktown LGA (Domain 9).

Age Groups	Allied Health and Other Services	Diagnostic tests	General Practitioner	Pharmaceutical Benefits Scheme	Public Hospital Admitted Patient	Public Hospital Emergency Department	Public Hospital Outpatient	Specialist Services	Total Services
20–24 years	918	10,314	10,273	11,704	11,261	3909	12,573	1921	62,873
25–34 years	962	9503	7776	13,244	8247	2698	13,701	1799	57,930
35–44 years	4245	31,252	22,219	58,707	32,066	7849	29,884	6559	192,781
45–54 years	10,386	64,706	39,420	126,292	85,551	12,962	46,475	14,141	399,933
55–64 years	3888	19,992	10,828	37,655	34,936	2864	14,506	5189	129,858
65 years and over	32,587	102,836	70,582	207,313	277,350	17,271	94,672	34,572	837,183
Total (inflation adjusted)	52,987	238,603	161,098	454,916	449,411	47,553	211,811	64,180	1,680,559

**Table A10:** Extra costs incurred due to LHL for people diagnosed with *Mental illness* in the Blacktown LGA (Domain 9).

Age Groups	Allied Health and Other Services	Diagnostic Tests	General Practitioner	Pharmaceutical Benefits Scheme	Public Hospital Admitted Patient	Public Hospital Emergency Department	Public Hospital Outpatient	Specialist Services	Total Services
20–24 years	31,629	1582	15,988	10,661	60,255	12,269	9747	8883	151,014
25–34 years	22,621	1506	11,438	10,297	45,868	6970	9022	6497	114,219
35–44 years	59,606	4490	31,685	40,317	123,883	17,408	26,990	20,565	324,944
45–54 years	79,245	8805	50,699	67,693	148,695	20,790	33,647	33,107	442,681
55–64 years	15,142	2706	12,270	14,540	27,325	3296	6138	7550	88,967
65 years and over	45,101	14,980	67,039	59,535	173,644	18,970	39,884	22,743	441,896
Total (inflation adjusted)	253,343	34,069	189,120	203,043	579,670	79,704	125,428	99,346	1,563,723

**Table A11:** Extra costs incurred due to LHL for people diagnosed with *Cancer* in the Blacktown LGA (Domain 9).

Age Groups	Allied Health and Other Services	Diagnostic Tests	General Practitioner	Pharmaceutical Benefits Scheme	Public Hospital Admitted Patient	Public Hospital Emergency Department	Public Hospital Outpatient	Specialist Services	Total Services
20–24 years	21	1660	2776	1837	6272	98	3716	2203	18,583
25–34 years	20	1757	2241	2615	6676	94	3712	2359	19,474
35–44 years	81	6087	7297	19,877	31,946	427	15,372	11,665	92,752
45–54 years	216	13,190	15,638	63,980	104,014	1435	47,687	36,152	282,312
55–64 years	84	4444	5247	26,902	43,910	612	20,262	14,891	116,352
65 years and over	801	26,729	38,427	196,165	311,814	4246	134,502	95,816	808,500
Total (inflation adjusted)	1223	53,868	71,625	311,375	504,633	6912	225,250	163,087	1,337,973

**Table A12:** Extra costs incurred due to LHL for people diagnosed with *Chronic kidney disease* in the Blacktown LGA (Domain 9).

Age Groups	Allied Health and Other Services	Diagnostic Tests	General Practitioner	Pharmaceutical Benefits Scheme	Public Hospital Admitted Patient	Public Hospital Emergency Department	Public Hospital Outpatient	Specialist Services	Total Services
20–24 years	10	2190	1400	1668	7001	2065	2077	577	16,988
25–34 years	10	2119	1064	1841	6265	1495	2265	551	15,610
35–44 years	42	5457	3024	8533	27,715	4341	6593	2128	57,833
45–54 years	111	9500	5350	19,391	78,629	8050	14,718	4597	140,346
55–64 years	45	2790	1471	5976	29,170	2210	5051	1612	48,325
65 years and over	450	14,933	9510	38,666	270,199	15,194	23,762	9836	382,550
Total (inflation adjusted)	669	36,988	21,819	76,074	418,980	33,354	54,467	19,303	661,653

**Table A13:** Extra costs incurred due to LHL for people diagnosed with *Respiratory disorders* in the Blacktown LGA (Domain 9).

Age Groups	Allied Health and Other Services	Diagnostic Tests	General Practitioner	Pharmaceutical Benefits Scheme	Public Hospital Admitted Patient	Public Hospital Emergency Department	Public Hospital Outpatient	Specialist Services	Total Services
20–24 years	34	1615	5752	5019	8993	2568	2746	1394	28,121
25–34 years	33	1364	4326	4085	5524	1426	2850	1399	21,007
35–44 years	139	4474	12,368	13,145	17,698	3935	6272	5527	63,558
45–54 years	365	9440	21,927	28,759	40,752	7861	10,031	11,694	130,829
55–64 years	147	2999	6038	11,251	16,963	2832	3180	3761	47,171
65 years and over	1476	16,163	38,836	100,374	172,376	30,324	19,563	19,421	398,533
Total (inflation adjusted)	2194	36,054	89,246	162,634	262,306	48,947	44,642	43,197	689,220

**Table A14:** Extra costs incurred due to LHL for people diagnosed with *Endocrine disorders* in the Blacktown LGA (Domain 9).

Age Groups	Allied Health and Other Services	Diagnostic Tests	General Practitioner	Pharmaceutical Benefits Scheme	Public Hospital Admitted Patient	Public Hospital Emergency Department	Public Hospital Outpatient	Specialist Services	Total Services
20–24 years	220	2930	4260	3018	3243	707	4285	648	19,311
25–34 years	217	2960	3240	4700	2839	370	4595	610	19,531
35–44 years	982	8295	9356	23,019	11,513	922	10,122	2175	66,384
45–54 years	2749	15,461	17,012	42,291	30,141	1447	18,794	4466	132,361
55–64 years	1129	4596	4853	19,189	11,470	350	6516	1548	49,651
65 years and over	9477	25,567	31,829	114,139	91,242	2132	35,104	9467	318,957
Total (inflation adjusted)	14,775	59,809	70,550	206,357	150,448	5927	79,416	18,914	606,196

**Table A15:** Extra costs incurred due to LHL for people with different chronic conditions—Method 2.

	Domain 6				Domain 9			
Age group	Diabetes	CVD	Mental Disorder	CKD	Diabetes	CVD	Mental Disorder	CKD
20–24 years	9536	22,804	31,788	17,399	3149	7531	10,497	5746
25–34 years	2707	9326	6796	6710	2707	9326	6796	6710
35–44 years	15,615	111,973	44,709	55,648	7702	55,232	22,053	27,449
45–54 years	21,068	285,080	47,475	416,764	14,961	202,443	33,713	295,956
55–64 years	11,218	258,025	16,136	340,744	4207	96,759	6051	127,779
65 years and over	6299	550,962	10,311	1,061,391	15,599	1,364,430	25,535	2,628,482
Total (inflation adjusted)	66,444	1,238,170	157,215	1,898,656	48,326	1,735,721	104,645	3,092,121
